# EBOLA in the Big Apple - how a single case paralyzed a city

**DOI:** 10.1186/2047-2994-4-S1-O15

**Published:** 2015-06-16

**Authors:** ML Tapper

**Affiliations:** 1 Division of Infectious Diseases, Lenox Hill Hospital, New York, NY, USA

## Introduction

A single case of Ebola virus disease (EVD) in a physician returning from service with MSF in Liberia set off widespread public anxiety. The physician was asymptomatic on his return but became febrile several days later, tested positive for EVD, and required admission to a specialized facility. In the interim, he had traveled widely to public places in New York before his diagnosis.

## Objectives

To describe the clinical, epidemiologic, public health and political aftermath of a single case of EVD in a physician returning from West Africa.

## Methods

Describe the events surrounding this physician's diagnosis, the media frenzy and political overreaction by state and local politicians including a confrontation between political leaders and public health authorities.

## Results

The extensive preparedness and training activities by public health authorities and health care facilities, including establishment of designated EVD diagnostic facilities and treatment centers were inadequate to overcome the ensuing public panic about potential Ebola transmission manifested by fear about riding the subways, visiting public sites or facilities where the physician was alleged to have wandered in New York prior to his diagnosis, and fears about seeking or providing treatment at the facility where the physician was hospitalized. There was avoidance of the large African community in New York, irrespective of their country of origin. The political ramifications included orders by two local governors to involuntarily restrain and isolate any additional healthcare providers returning from Ebola endemic areas which had a chilling effect on recruitment efforts for replacement workers

## Conclusion

A single case of Ebola Virus disease in a returned healthcare worker caused widespread panic despite educational efforts by public health officials and healthcare facilities. These efforts were inadequate to overcome general distrust of the government, media frenzy, and initial misstatements by federal health officials. Considerable harm was also done by overzealous political figures anxious to use the public's concerns to advance their own political agendas.

## Disclosure of interest

None declared.

